# Functional Evaluation of a Force Sensor-Controlled Upper-Limb Power-Assisted Exoskeleton with High Backdrivability

**DOI:** 10.3390/s20216379

**Published:** 2020-11-09

**Authors:** Chang Liu, Hongbo Liang, Naoya Ueda, Peirang Li, Yasutaka Fujimoto, Chi Zhu

**Affiliations:** 1 Department of Environment and Life Engineering, Maebashi Institute of Technology, 460-1 Kamisadori, Maebashi, Gunma 371-0816, Japan; m1756503@maebashi-it.ac.jp (C.L.); m1656504@maebashi-it.ac.jp (H.L.); m1956502@maebashi-it.ac.jp (N.U.); m2056504@maebashi-it.ac.jp (P.L.); 2 Department of Electrical and Computer Engineering, Faculty of Engineering, Yokohama National University, 79-5 Tokiwadai, Hodogaya-ku, Yokohama 240-8501, Japan; fujimoto@ynu.ac.jp; 3 Department of Systems Life Engineering, Maebashi Institute of Technology, 460-1 Kamisadori, Maebashi, Gunma 371-0816, Japan

**Keywords:** upper-limb, power-assisted exoskeleton, backdrivability, workspace

## Abstract

A power-assisted exoskeleton should be capable of reducing the burden on the wearer’s body or rendering his or her work improved and efficient. More specifically, the exoskeleton should be easy to wear, be simple to use, and provide power assistance without hindering the wearer’s movement. Therefore, it is necessary to evaluate the backdrivability, range of motion, and power-assist capability of such an exoskeleton. This evaluation identifies the pros and cons of the exoskeleton, and it serves as the basis for its subsequent development. In this study, a lightweight upper-limb power-assisted exoskeleton with high backdrivability was developed. Moreover, a motion capture system was adopted to measure and analyze the workspace of the wearer’s upper limb after the exoskeleton was worn. The results were used to evaluate the exoskeleton’s ability to support the wearer’s movement. Furthermore, a small and compact three-axis force sensor was used for power assistance, and the effect of the power assistance was evaluated by means of measuring the wearer’s surface electromyography, force, and joint angle signals. Overall, the study showed that the exoskeleton could achieve power assistance and did not affect the wearer’s movements.

## 1. Introduction

Owing to the accelerated aging of the world’s population, the average age of heavy-duty workers in environments such as construction sites, logistic centers, and nursing care facilities is steadily increasing. Therefore, the development and adoption of power assistance devices to alleviate the burdens of these workers are becoming necessary and critical. One such power assistance device is an exoskeleton, a mechanical structure that can be worn over the user’s body, and it can effectively reduce the wearer’s burden by transferring the load to itself. Thus, the research and development pertaining to exoskeletons has recently received significant attention [1].

An ideal exoskeleton should assist the wearer, be convenient to use, and not induce feelings of restraint or discomfort in the wearer. However, to ensure that the active joints of the exoskeleton possess the necessary driving torque, a reducer with a large reduction ratio is often employed, which leads to the loss of backdrivability [2]. In other words, the active joints of the exoskeleton cannot be driven by the external forces when no control is applied. This not only causes inconvenience when wearing and taking off the exoskeleton, but it also restricts the wearer’s movements in unexpected situations, such as power failure or loss of control. This in turn results in a strong sense of restraint and poses certain safety risks. Therefore, the determination of whether an exoskeleton’s active joints possess backdrivability is an important factor affecting the comfort and safety that the exoskeleton can provide [3,4]. The active joints of the exoskeleton developed in this study ensures that backdrivability is incorporated to improve the convenience and comfort of the exoskeleton and ensure the wearer’s safety.

When the degrees of freedom (DOF) required for an exoskeleton are being determined, the most important criterion is the range of motion for each human joint. Therefore, the kinematic measurement and the analysis of human limb activities form the basis for developing or optimizing the exoskeleton design and control methods [5,6,7]. However, an exoskeleton is generally a rigid structure that is worn on the outside of the body, and its motion is restricted by that of the wearer. Moreover, the workspace after an exoskeleton is worn is affected by body shape, athletic ability, and other personal differences. Therefore, the level of impact needs to be accurately analyzed to evaluate and improve the exoskeleton design. At present, the DOF evaluation of an exoskeleton is mostly focused on measuring and comparing the rotatable angle of each joint with the range of motion of a human joint in the same direction [7,8,9]. However, it is difficult to properly conduct the evaluation of the workspace under the multi-DOF coordinated motion via this approach. Thus, this study uses a motion capture system to measure and analyze the range of motion of the shoulder and elbow joints in the workspace, before and after the exoskeleton is worn. Thereafter, the results are used to evaluate whether the exoskeleton meets the wearer’s needs for the workspace.

In the control of exoskeleton, physiological or biomechanics signals are mainly used to speculate the motion intention of wearer and achieve power assisting. Within physiological signals, the sEMG signal, with relatively obvious features, is often used as the control signal of the exoskeleton [10,11,12]. However, because of large differences between wearers, the existence of multi-joint muscles causes the speculation of motion intention to be complicated [13]. Therefore, the fuzzy control system [14,15] can simplify the complexity of system design. For example, Neural-Fuzzy control [10] is based on the neural network to improve the influence of individual differences in the exoskeleton control. However, the sEMG signal is susceptible to the influence of sweat and muscle fatigue, which renders application in actual work scenarios challenging. On the other hand, biomechanical signals are measured by limb’s kinematic and dynamic properties. The biomechanical signals have more obvious features and clearer physical meanings. For example, some exoskeletons with biomechanical signals use the threshold control [16], admittance control [17], and PID control [18] to implement control. Among them, PID control advantaged in computational load, control accuracy, simplicity, and robustness have been considered suitable for exoskeleton [19]. On the other hand, controlling the exoskeleton through a torque sensor [20], requires that the sensors be installed on all active joints, which increases the weight and cost of the exoskeleton. For the force-sensitive resistors (FSRs) [16], there exists a response time lag, and it is difficult in this case to achieve measurement accuracy; hence, it is difficult to use the FSRs for real-time power-assist control. Therefore, in this study, a small and compact three-axis strain-gauge force sensor was fixed on the extremity of the exoskeleton, and the signals of the wearer’s forces on the exoskeleton were read and the power assistance were achieved. This approach effectively avoids the influences of wearers’ individual differences on the control, and it also saves on time and costs that are required for assembling electrodes or sensors. Furthermore, heart rate [21], oxygen consumption [22], and the sEMG signal [23,24] can be used to evaluate the power assistance from different standpoints. These methods all require different external equipment for assistance, and the requirements for the experimental environment also vary among one another. In this study, control was achieved by fixing the force sensors on the exoskeleton. In this manner, we were able to compare the force signal with the weight of the load in the most convenient and effective way to confirm the power assistance. Furthermore, the sEMG signals were used to verify the effect of power assistance.

In summary, this study introduces the developed upper-limb power-assisted exoskeleton with backdrivability, which can satisfy the wearer’s needs as he or she performs daily movements. It is believed that, when daily tasks are performed, movements such as flexion or extension (lifting and lowering) impose the greatest burden on the carrier. Therefore, the upper-limb power-assisted exoskeleton developed in this study primarily aims to provide power assistance for the flexion/extension of the shoulder and elbow joints. In the flexion/extension direction of the exoskeleton, each active joint was designed to be powered by a brushless direct current (BLDC) motor with two-stage reduction (timing-belt pulley set and special gear reducer), and it is observed to possesses the desired backdrivability whilst providing power assistance. The exoskeleton was also designed with multiple passive joints to support movements other than flexion/extension. The workspace before and after the exoskeleton was worn (i.e., without and with the exoskeleton) was measured and analyzed, and the results verified that the DOF design of the exoskeleton can meet the needs of daily life. The control system of the exoskeleton is based on force sensor. The power assistance experiments are to carry or lift an object weighting 10 kg onto the 1.2 m and 1.8 m high. In particular, the heavy object had to be lifted above the head so it could be carried to a height of 1.8 m, which posed high requirements for the workspace and the control coordination of the exoskeleton. The force signals and sEMG signals obtained during the carrying process verified that the exoskeleton could achieve effective power assistance. It was also clarified from the action angles that the control method did not require a specific working trajectory, and power assistance was achieved completely as per the motion intention of the wearer.

The remainder of this paper is organized as follows. Section 2 introduces the structural design and the backdrivability of the exoskeleton, and Section 3 introduces the designed workspace of the exoskeleton. Moreover, it also describes the measurement and analysis of the workspace before and after the exoskeleton was worn for evaluation of the exoskeleton DOF design. Section 4 introduces the control method based on the force sensor as well as the evaluation of the power-assist capability of the exoskeleton. Finally, Section 5 and Section 6 present the discussion and conclusions, respectively.

## 2. Design of the Exoskeleton

### 2.1. Structural Design of the Exoskeleton

**Figure 1:**
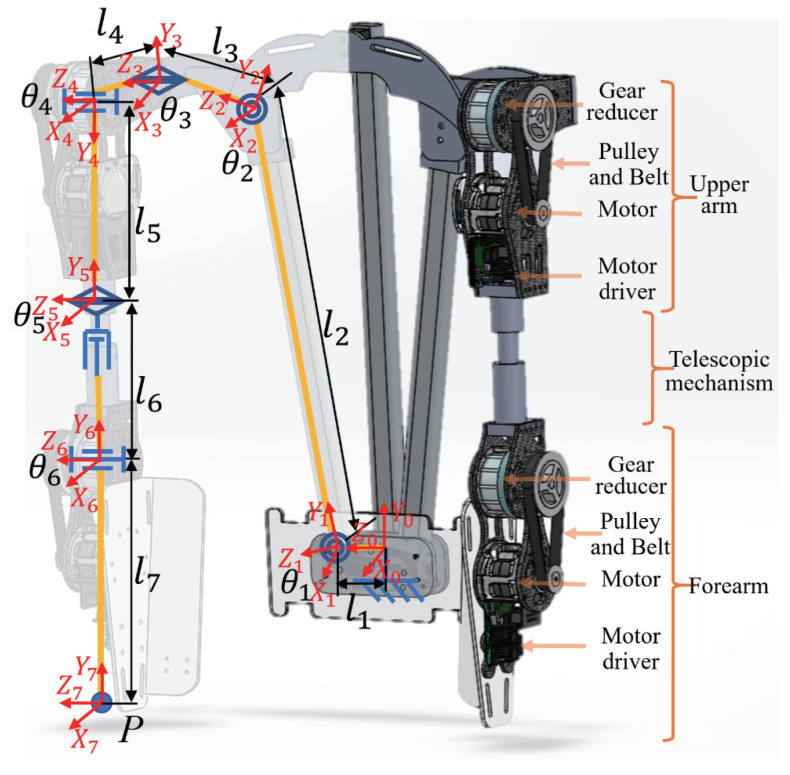
Mechanical structure of the exoskeleton.

The exoskeleton was designed on the basis of the DOF of the human shoulder and elbow joints, and its overall design is shown in Figure 1. A single arm of the exoskeleton consists of six rotary joints and a telescopic mechanism. The specific design parameters are listed in Table 1. The combination of $\theta_{1}$, $\theta_{2}$, and $\theta_{3}$ corresponds to the abduction/adduction of the shoulder, $\theta_{4}$ corresponds to the flexion/extension of the shoulder, $\theta_{5}$ corresponds to the internal/external angle of the shoulder, and $\theta_{6}$ corresponds to the flexion/extension of the elbow. Finally, $\theta_{4}$ and $\theta_{6}$ correspond to the active joints, which can provide power assistance to the wearer. The others are passive joints, which can follow the wearer’s movements, and do not affect the wearer’s free movement. With the combination of active and passive joints, the exoskeleton could exhibit the necessary power-assist capability; thus, the number of motors was reduced, and the cost and weight were controlled. In the exoskeleton, the motors and reducers accounted for most of the weight. Carbon fiber materials were employed to fabricate the main structural parts to lower the weight; thereby, the weight of the two-arm prototype was reduced to 5.1 kg (excluding the weight of the batteries).

The exoskeleton could also be adapted to the shoulder width by the passive joint ($\theta_{1}$) and the upper-arm length (from shoulder joint to wrist joint) via the telescopic mechanism of $l_{6}$ for different wearers.

### 2.2. Design of Active Joints with Backdrivability

**Figure 2:**
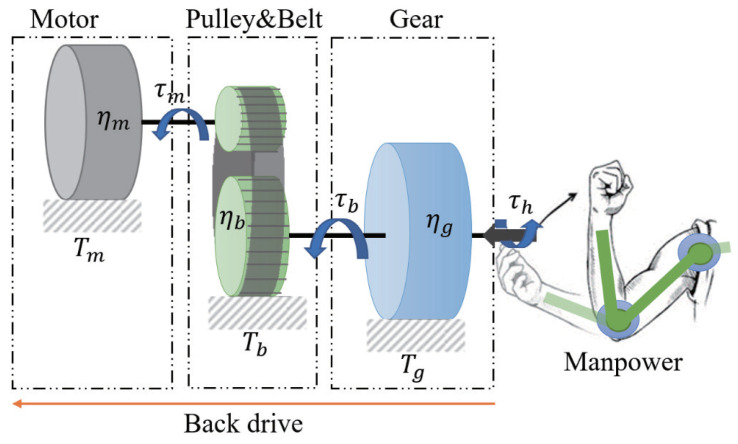
Schematic of the active joint.

The exoskeleton (Figure 1) uses flat BLDC motors (EC45flat-70W, Maxon Motor Co., Sachseln, Switzerland) as the actuators, which deliver the output to the active joints of the exoskeleton after two-stage reduction. This setup consists of a timing-belt pulley set red (3:1) and a gear reducer (100:1). The corresponding schematic is shown in Figure 2. This approach facilitated achievement of a high reduction ratio and simultaneously realized the parallel assembly of the motor and reducer through the transmission of the timing-belt pulley set; thus, the thickness of the active joints and the space occupied by the exoskeleton outside the wearer were effectively reduced. As a result, the exoskeleton can better fit the wearer’s body, improving comfort while it is worn, and it is easier to control, debug, and maintain.

Backdrivability is a critical criterion for wearable exoskeletons. It is a measure of how easily a torque applied on the output axis inversely drives the input axis and the motor [25]. Therefore, for the exoskeleton to be driven backward, the wearer has to overcome the rotation resistance of the exoskeleton and drive the exoskeleton to move. In other words, large rotation resistance impairs the backdrivability.

During backdriving, the gear reducer and timing-belt pulley can increase the speed in the system. Their backdrive transmission efficiencies are $\bar{\eta_{g}}$ and $\bar{\eta_{b}}$, and their reduction ratios are $G_{g}$ and $G_{b}$, respectively. When the wearer applies a torque ($\tau_{h}$) to the output shaft of the active joint (the output shaft of the gear reducer), the torque is transmitted through the gear reducer, and a torque ($\tau_{b}$) is generated to drive the timing-belt pulley. This in turn generates a torque ($\tau_{m}$) to the motor shaft after it passes through the timing-belt pulley set. During backdrive, the torques $\tau_{h}$, $\tau_{b}$, and $\tau_{m}$ on each rotating shaft overcome the friction torques $T_{g}$, $T_{b}$, and $T_{m}$ of the gear reducer, timing-belt pulley, and motor, respectively, to perform backdrive. The required input torque $\tau_{h}$ can be expressed as follows:

(1) $$\tau_{h}=T_{g}+\frac{T_{b}G_{b}}{\bar{\eta_{b}}}+\frac{T_{m}G_{m}G_{b}}{\bar{\eta_{g}}\bar{\eta_{b}}}$$

It can be observed from Equation (1) that, in order to enhance the backdrivability via reduction in $\tau_{h}$, lowering the friction torque and reduction ratio and improving the backdrive efficiency are effective approaches. However, under normal circumstances, to ensure the output torque of the exoskeleton, a high reduction ratio is required. Therefore, the most direct and feasible approach is to improve the backdrive efficiency of the transmission system and reduce the friction torque.

In the current system, the friction torque of the motor and the timing-belt pulley set ($T_{m}$ and $T_{b}$) are 4.9 mN·m and 1.0 mN·m, respectively. Despite the values being small, these parameters are amplified by the reducer when driven backward. Because of its high transmission efficiency (96.0%) and small friction torque, the timing-belt pulley set does not have a significant influence on the backdrivability when used for the first-stage reduction. However, the maximum reduction ratio the pulley can provide is limited by its diameter. Thus, it is critical to choose a suitable gear reducer for the second-stage reduction. In this study, a newly developed bilateral drive gear [26] with high backdrivability was selected, and its reduction ratio is 100:1.

Herein, we compared the backdrivability of three different forms of gear reducers, namely, harmonic drive gears (CSF-17-100-2A-GR, Harmonic Drive Systems Inc., Tokyo, Japan), hybrid reducers (LGU75-4MLD/5MLG, Matex Co., Ltd., Osaka, Japan) and bilateral drive gears. They have similar shapes and the same reduction ratio. The harmonic drive gear is a gear reducer commonly used in robotic mechanisms. The hybrid reducer is a traditional planetary gear reducer. It has the advantage of combining any reduction ratio as needed.

The torque required to drive the following three groups of parts with the external torque was measured, respectively:

(1) The gear reducer only ($T_{g}$).
(2) The gear reducer and the timing-belt pulley ($T_{g}+\frac{T_{b}G_{b}}{\bar{\eta_{b}}}$).
(3) The gear reducer, timing-belt pulley, and motor, that is, the entire active joint ($T_{g}+\frac{T_{b}G_{b}}{\bar{\eta_{b}}}+\frac{T_{m}G_{m}G_{b}}{\bar{\eta_{g}}\bar{\eta_{b}}}$).

In measurements, a digital spring scale is hooked on the part connected output axis of the gear reducer and is pulled gradually until its force is big enough to make the axis start up rotation. This torque of starting up rotation is considered as the backdrive torque of the gear reducer that is the product of multiplying the force by the length from the axis to the hooking point of the digital spring scale. Similarly, three different types of gear reducers (bilateral drive gear, hybrid reducer and harmonic driver gear) are installed into the active joint of the exoskeleton in sequence, and their corresponding backdrive torque is measured, respectively.

**Figure 3:**
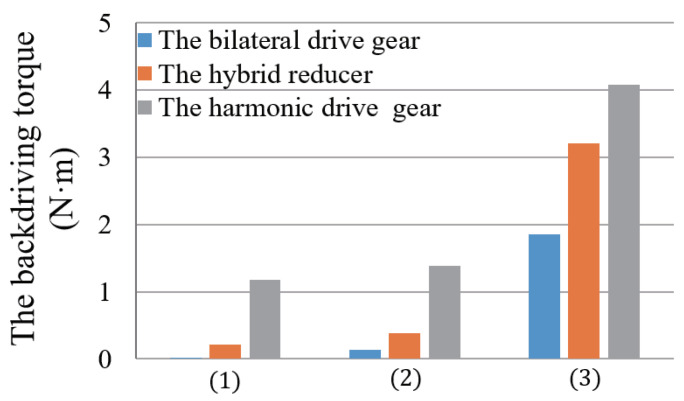
Measured backdriving torques of three different forms of gear reducers used for the active joint: (1) The gear reducer only. (2) The gear reducer and the timing-belt pulley. (3) The gear reducer, timing-belt pulley, and motor, that is, the entire active joint.

The measurement results are shown in Figure 3. The harmonic drive gear has the advantage of no backlash; however, owing to its elastic deformation characteristic, the torque required for the backdrive was as high as 1.17 N·m, and the backdrive efficiency was 54.6%. This largely reduced its backdrivability. For the hybrid reducer, a reduction ratio of 100:1 was adopted by combining three independent planetary gears (4:1, 5:1, 5:1). Although the torque required for backdrive was only 0.21 N·m, the connection transmission of multiple independent planetary gears affected the efficiency, and the backdrive efficiency was 67.4%, which also notably lowered the overall backdrivability. Finally, it can be observed that the bilateral drive gear exhibited clear advantages during backdrive. The starting torque required for backdrive was only 0.02 N·m, and the forward drive and backdrive efficiencies were 89.0% and 85.3%, respectively. The high efficiency and minimal friction torque during backdrive laid the foundation for reducing the starting torque of the active joint to 1.85 N·m when it was driven backward.

The active joint of the exoskeleton was designed in a two-stage reduction form, which effectively controlled the thickness of the active joint and ensured the high backdrivability while achieving a high reduction ratio (300:1). Thus, the wearer can easily drive the exoskeleton to move when it is not under control. Furthermore, the active joint renders the exoskeleton more convenient to wear and take off, which greatly improves the comfort and safety provided to the wearer.

## 3. Workspace of the Exoskeleton

The backdrivability guarantees the flexibility of the active joints when the exoskeleton is not under control. However, further verification is required to determine whether the joint design of the exoskeleton can meet the wearer’s needs for the workspace. This section describes the evaluation of the designed workspace of the exoskeleton and the wearer’s actual workspace before and after the exoskeleton is worn.

### 3.1. Measurement of the Exoskeleton’s Workspace

First, according to the exoskeleton’s single-arm DOF, shown in Figure 1, and the actual design parameters listed in Table 1, the Denavit–Hartenberg method [27] was applied to calculate the designed workspace of the exoskeleton.

According to Japanese statistics for Japanese young male’s body dimensions data [28], we set the arm and forearm length of the exoskeleton to 0.56 m and 0.25 m, respectively. The workspace of the exoskeleton was calculated using the reachable coordinates of wrist position (P in Figure 1) during movement of the shoulder and elbow joints.

These two position coordinates constructed the boundary surfaces in a three-dimensional (3D) space, the areas of which can be used to represent the size of the designed workspace. The results can be expressed as hemispherical surfaces with areas of 2.25 m${}^{2}$ and 0.28 m${}^{2}$, in which the exoskeleton shoulder and elbow joints were the focuses of the two hemispherical surfaces, respectively.

However, the actual workspace of the exoskeleton wearer is restricted by the wearer’s body and the exoskeleton mechanical structure. It is also affected by the wearer’s body shape, joint flexibility, and other personal differences existing among multiple wearers. Therefore, it is necessary to further measure and analyze the actual workspace after the wearer puts on the exoskeleton.

Wearable exoskeletons are a multiple-DOF system to ensure that the wear’s movements are not hindered. The movement of the human shoulder complex is controlled by the tendons and ligaments around the joints, which determine the different ranges of motion for each individual [29]. Furthermore, the range of motion has an irregular shape, making it very difficult for an exoskeleton to mimic the motion of the shoulder joint. Unlike in the case of a single-DOF joint, although the motion range for a multi-DOF joint can be evaluated by measuring its rotatable angles in each direction, the range of the multi-DOF coordinated motion in 3D space cannot be fully evaluated. Movements of different DOFs directly affect how the exoskeleton follows the wearer’s movement, which affects the wearer’s perception or limits the range of motion. Therefore, we considered that a direct measurement and comparison of the workspaces before and after the exoskeleton is worn can realize a more accurate evaluation of the DOF of the exoskeleton.

**Figure 4:**
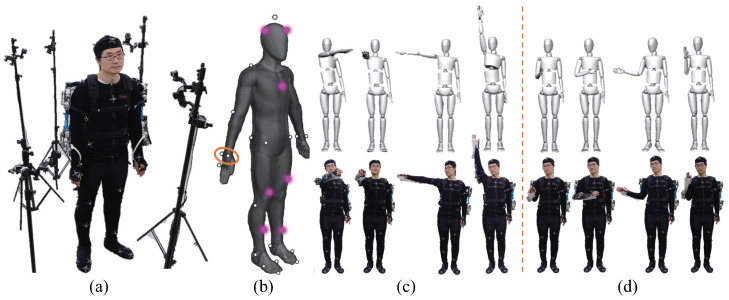
(**a**) experimental setup and overview of the motion capture; (**b**) marker locations; (**c**) measurement of the shoulder joint workspace; (**d**) measurement of the elbow joint workspace.

In this study, the OptiTrack motion system (Natural Point, Inc., Corvallis, OR, USA), which is an optical motion capture system based on marker tracking, was adopted to conduct the measurement. It uses six cameras that emit infrared light and receive the reflected light from the spherical markers to create a static workspace (Figure 4a). The coordinates of the markers were recorded at a sampling frequency of 120 Hz.

For each wearer, 42 spherical markers were placed over his or her body (the marker locations are shown in Figure 4b). Through the accompanying analysis software, a human body model can be established, and the position, velocity, and acceleration data of the markers can be obtained. One of the purposes of establishing the human body model is to obtain the displacement of the wearer’s torso during the measurement process, which is used to reduce the measurement error of the workspace. A spherical marker was placed on each side of the wrist, and its midpoint was used to represent the position of the wrist joint in space. The workspace of the shoulder and elbow joints were measured and analyzed before and after the exoskeleton was worn. This can help verify the design of the exoskeleton joints and evaluate the rationality of the design. In the experimental results, the workspace of the shoulder and elbow joints was represented by the areas that are swept by the wrist joint in space during movement. Measurement of the shoulder joint workspace is shown in Figure 4c, and the wearer’s elbow joint remained fully extended during the measurement. Meanwhile, the shoulder joint repeatedly moved in the movable space so that all the reachable positions of the wrist could be covered, and the wrist was moved to the maximum extent. Measurement of the elbow joint workspace is shown in Figure 4d, and the flexion and extension of the elbow joint are often accompanied by the internal/external rotation of the forearm. Therefore, the upper arm remained still during the measurement, and the forearm repeatedly moved in the movable space so that all the reachable positions of the wrist could be covered, and the wrist was moved to the maximum extent.

The trajectories of experiments were recorded by the motion capture system, respectively. Thus, the coordinate sets of the reachable positions for the wrist joint during the movement of the shoulder and elbow joints could be obtained. Thereafter, the boundary areas of the position coordinates were constructed in the 3D space, and the areas were calculated to represent the workspaces corresponding to the joints.

### 3.2. Evaluating the Exoskeleton’s Workspace

#### 3.2.1. Comparison of the Workspace before and after the Exoskeleton Was Worn

**Figure 5:**
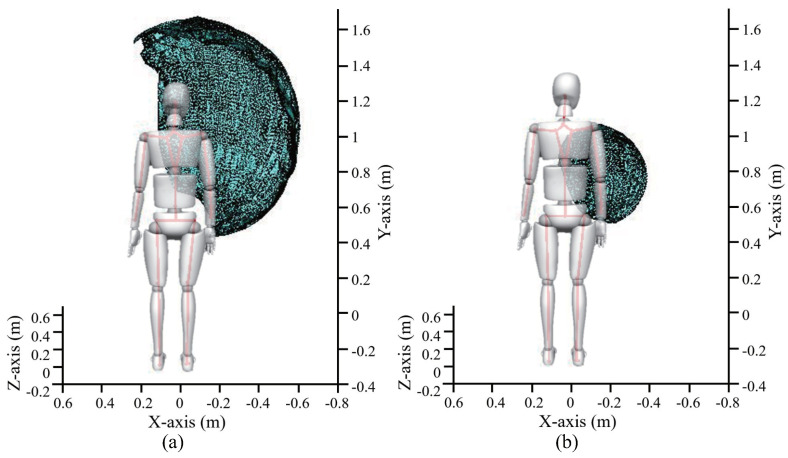
Boundary area of the shoulder joint (**a**) and the elbow joint (**b**) constructed by workspace measurement results of subject A after the exoskeleton was worn.

As per the experimental methods described above, the workspaces of the shoulder and elbow joints before and after the exoskeleton was worn were measured for four subjects with different heights and arm lengths. The height and arm length of subject A were close to the average values provided by Japanese statistics, and those of subject B were less than the average values, and those of subjects C and D were above the average values. Figure 5a,b show the boundary area of the shoulder joint and the elbow joint constructed by using workspace measurement results of subject A after the exoskeleton was worn, respectively. Table 2 and Table 3 present the measurement results of the four subjects.

On comparing the designed workspaces of the exoskeleton, given in Section 3.1, (Shoulder: 2.25 m${}^{2}$; Elbow: 0.28 m${}^{2}$), it can be observed that the mechanical structure of the exoskeleton and the wearer had a mutual influence on one another during movement. Although the designed workspace of the exoskeleton is larger than the wearer’s workspace, it is not appropriate for use in evaluating the exoskeleton design alone. The results show the workspaces of the four subjects after the exoskeleton was worn were slightly smaller than those measured before the exoskeleton was worn. However, the workspace of more than 80% could be reached than that before the exoskeleton was worn. Thus, the results show that wearing the exoskeleton did not affect the wearer’s range of motion significantly.

#### 3.2.2. Comparison with the Workspace Required for Daily Life

**Figure 6:**
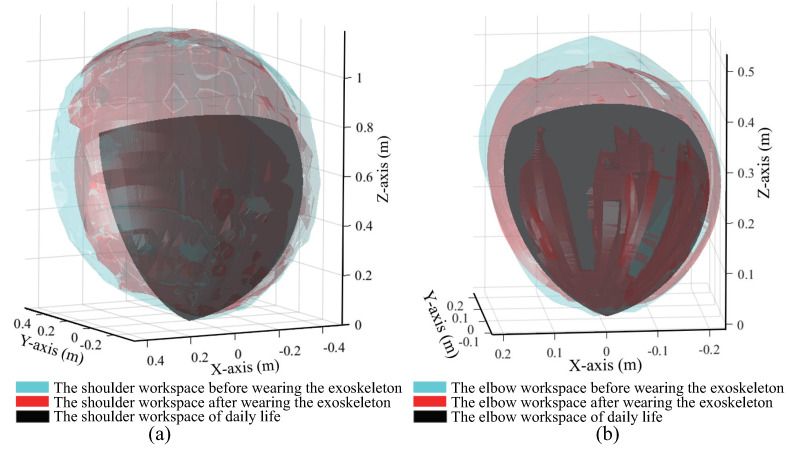
The results of the subject A’s workspace of daily life, before, and after the exoskeleton was worn of shoulder joint (**a**) and elbow joint (**b**). The workspace before the exoskeleton was worn (blue), contains the workspace after the exoskeleton was worn (red), and the latter further contains the commonly used space for daily life (black).

Furthermore, the workspaces of the upper limbs that are frequently active for daily life were calculated and compared with the measurement results obtained as described in the previous subsection. The common motions of people exhibited in daily life were measured [30]. For example, drinking from a glass requires the elbow joint to move $121^{{}^{\circ}}$ in the direction of flexion/extension. In another example, applying perfume on the opposite side of the body requires the shoulder joint to move $109^{{}^{\circ}}$ and $99^{{}^{\circ}}$ in the direction of flexion/extension as well as the direction of abduction/adduction, respectively. On the basis of the maximum angles of the joints attained during daily movements, the commonly used workspaces were calculated using the human upper limb motion ranges. The ranges of motion for the shoulder (flexion/extension: $-53^{{}^{\circ}}$ to $109^{{}^{\circ}}$; abduction/adduction: $0^{{}^{\circ}}$ to $105^{{}^{\circ}}$; internal/external: $-53^{{}^{\circ}}$ to $63^{{}^{\circ}}$) and elbow joints (flexion/extension: $0^{{}^{\circ}}$ to $121^{{}^{\circ}}$), as well as subject A’s arm length and forearm length, were used for the calculation. The workspaces commonly used by the shoulder and elbow joints were obtained to be 0.67 m${}^{2}$ and 0.17 m${}^{2}$, respectively. These calculated results were compared with subject A’s measurement results obtained as described in the previous subsection. As shown in Figure 6, the workspace before the exoskeleton was worn (blue), contains the workspace after the exoskeleton was worn (red), and the latter further contains the commonly used space for daily life (black). It can be observed from the results that the workspaces of the shoulder and elbow joints after the exoskeleton was worn completely covered the space required for daily life.

Although the workspace before the exoskeleton was worn was not fully covered, the uncovered parts were located on the edge and were not within the required workspaces for daily life. Therefore, it is considered that the workspace after the exoskeleton was worn satisfies the needs of daily life.

## 4. Evaluation of the Power Assistance of the Exoskeleton during Object Carrying Tasks

### 4.1. Control Method

**Figure 7:**
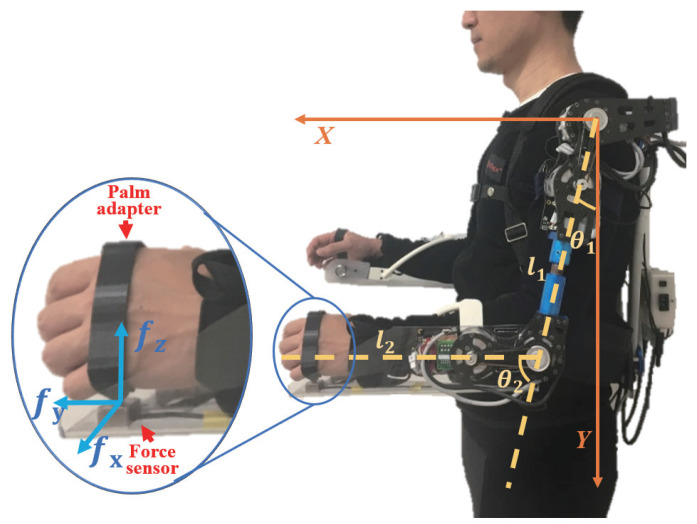
Position and measurement method of the force sensor.

As shown in Figure 7, a 3D-printed palm adaptor was used to connect the wearer’s hand with the force sensor, which was also the only point connected to the exoskeleton. A small and compact three-axis strain gauge force sensor (USL06-H12-500N-AP, Tec Gihan Co., Ltd., Kyoto, Japan) was fixed directly on the palm adapter under the palm of the wearer to detect the force components in the three horizontal and vertical directions generated on the contact surface. Thus, the force sensor could detect the force applied by the wearer on the exoskeleton with high sensitivity. Furthermore, the palm adaptor could transfer the force to the force sensor without hindering the flexibility and dexterity of the fingers, which could maintain the object in balance. The weight of the object was first transferred to the exoskeleton, which was shared by the exoskeleton and the wearer. The ratio of the weight directly borne by the exoskeleton to the weight of the object is the power-assist ratio.

The exoskeleton only provides power assistance to the flexion/extension action; thus, the force components ($f_{z}$, $f_{y}$) of the force sensor on the plane of flexion/extension were transformed into the force components ($F_{x}$, $F_{y}$) in the world coordinate system using Equation (2):

(2) $$\left[\begin{array}{c} F_{x}\\ F_{y} \end{array}\right]=\left[\begin{array}{cc} C_{12} & S_{12}\\ -S_{12} & C_{12} \end{array}\right]\left[\begin{array}{c} f_{z}\\ f_{y} \end{array}\right]$$

where $C_{12}=\cos\left(\theta_{1}+\theta_{2}\right)$, $S_{12}=\sin\left(\theta_{1}+\theta_{2}\right)$.

Furthermore, the force signals were converted into the torque signals ($\tau_{e}$ and $\tau_{s}$) of the elbow and shoulder joints for the wearer’s exoskeleton by using Equation (3) and the Jacobian matrix (Equation (4)):

(3) $$\left[\begin{array}{c} \tau_{e}\\ \tau_{s} \end{array}\right]=J_{aco}^{T}\left[\begin{array}{c} F_{x}\\ F_{y} \end{array}\right]$$

(4) $$J_{aco}^{T}=\left[\begin{array}{cc} l_{2}C_{12} & l_{2}S_{12}\\ l_{1}C_{1}+l_{2}C_{12} & -l_{1}S_{1}-l_{2}S_{12} \end{array}\right]$$

where $C_{1}=\cos\theta_{1}$, $S_{1}=\sin\theta_{1}$.

The angle information for $\theta_{1}$ and $\theta_{2}$ was obtained from the encoder of the motor in the active joint (denoted as $\theta_{1}$ and $\theta_{2}$ for convenience, which corresponds to $\theta_{4}$ and $\theta_{6}$ in Figure 1). The obtained torques, namely, $\tau_{e}$ and $\tau_{s}$, were used to control the force as follows:

(5) $$\begin{array}{c} \tau_{ed}=A_{e}\tau_{e}+\tau_{0}\\ \tau_{sd}=A_{s}\tau_{s}+\tau_{1} \end{array}$$

where $\tau_{0}$ and $\tau_{1}$ are the torques required to support the weight of the exoskeleton, obtained from the joint angle and the weight of the exoskeleton. The torques $\tau_{ed}$ and $\tau_{sd}$ tuned by coefficients $A_{e}$ and $A_{s}$ were used to control the output torque of the elbow and shoulder of the exoskeleton, respectively.

In the development of exoskeletons, it is very important to determine the power-assist ratio. However, the balance of the power-assist ratio and weight of the exoskeleton is a trade-off problem, due to the required high output torque of the actuator when the power-assist ratio increases. This also implies the increment of size and weight of the actuator. Thus, difficulties still remain in lightening and comforting the exoskeleton. On the contrary, since too small power-assist ratio will limit the application of the exoskeleton, we set the target power-assist ratio of the exoskeleton to 50%.

**Figure 8:**
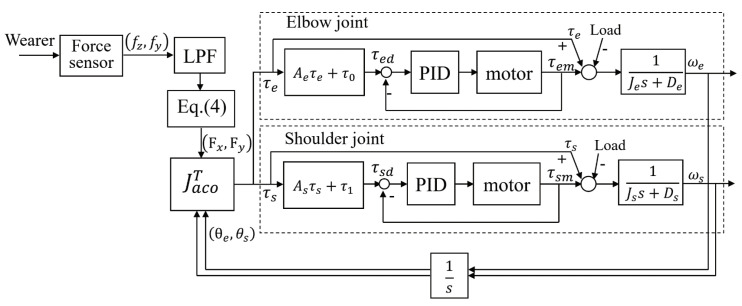
Block diagram of the control system for the exoskeleton.

Figure 8 displays the block diagram of the control system. The output torque of the active joint is transmitted to the object through the exoskeleton in order to achieve power assistance. From the equation, the coefficients $A_{e}$ and $A_{s}$ were used to tune the output torque and adjust the power-assist ratio of the exoskeleton. Simultaneously, the force applied by the wearer on the force sensor is also transmitted to the object through the exoskeleton. The power assistance can be realized through the joint action of the wearer and the exoskeleton.

Therefore, the exoskeleton needs to bear half of the load to achieve the target power-assist ratio of 50%. According to Equation (5), the target torque ($\tau_{ed}$ and $\tau_{sd}$) of the motor is equal to $\tau_{e}$ and $\tau_{s}$ of wearer after the amplification of the reducer (active joint) when $A_{e}$ and $A_{s}$ are set to 1. This means that the wearer and the exoskeleton each bear half of the weight of the load to achieve the target power-assist ratio of 50%. The PID control compensates the actual output torque based on the monitored current of the motor. According to the continuous torque (128 mN·m) of the motor and the reduction ratio (300:1), the maximum theoretical output torque of a single active joint is 38.4 N·m. The two arms of exoskeleton can support the elbow joint up to a weight of 30.7 kg by calculated as the length of the forearm is equals 0.25 m. For the shoulder joint, the exoskeleton can support a weight of 13.7 kg by calculated as the length of the arm equals 0.56 m. However, the elbow joint is rarely fully extended in actual carrying motion, which means the exoskeleton is able to load objects more than 13.7 kg.

**Figure 9:**
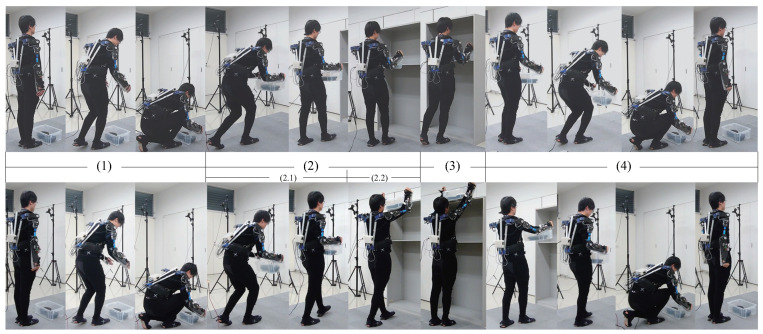
The experimental task is to carry a load from ground onto different heights (upper row is 1.2 m, bottom row is 1.8 m).

In view of the ethical considerations determined by the Ethics Committee of the Maebashi Institute of Technology, the subjects were fully informed and requested for consent before the experiments were conducted, and all required safety measures were taken including the emergency stop switch of the system, controlled by one person other than the operator and the subjects. Comparison experiments were conducted between the groups without the exoskeleton and with the exoskeleton via the control method described above. The subject was instructed to carry an object weighing 10 kg onto the different floors of a shelf at 1.2 m and 1.8 m high, and then carry it away to the floor of the ground from the shelf. Figure 9 shows the experiment in which the subject was wearing the exoskeleton. A height of 1.2 m is the common height to which items are placed during daily activities. The corresponding action is mainly completed through the movement of the elbow joint. The height of 1.8 m is the greatest height to which items can be placed without climbing assistance. The corresponding action is mainly completed through the coordinated control of the shoulder and elbow joints. Note that, as the weight of the heavy objects being carried in the experiment was evenly distributed, the object was lifted by both hands together with the same movement. Therefore, the data in the study were all measured from the right hand.

### 4.2. Experiment of Carrying a Heavy Object to the 1.2 m High

To perform the lifting action (flexion/extension of the elbow and shoulder), the work is mainly performed by the biceps and deltoids [31]. Therefore, their myoelectric potentials show the most noticeable changes during the lifting action, which are suitable for evaluating the load on the arm.

In this experiment, the Bagnoli sEMG Sensor (DELSYS Inc., Natick, MA, USA) was used to measure the sEMG signals. An electromyograph was designed with a built-in gain of $10^{4}$ V/V, a built-in filter with a 20 to 450 Hz bandwidth range and 12 dB/Oct attenuation. The raw sEMG signals have an output range of ±5 V. After the full-wave rectification of the raw sEMG signals, a 50-point moving average and 10 Hz low-pass filtering were applied to obtain the processed sEMG signals, which were used to represent the load on the relevant muscles.

The carrying action can be divided into four stages (shown in upper row in Figure 9):

(1) Bending over to pick up the heavy object (stage (1) in Figure 9).
(2) Lifting the heavy object, standing up, and approaching the shelf (stage (2) in Figure 9).
(3) Placing the heavy object on the 1.2 m high (stage (3) in Figure 9).
(4) Lifting the heavy object, backing up, bending over, and placing it back on the ground (stage (4) in Figure 9).

**Figure 10:**
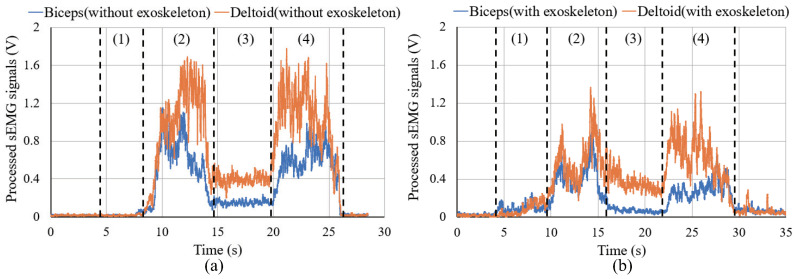
sEMG signals for carrying a load to the 1.2 m high for subject A. (**a**) is the results of sEMG signals of biceps and deltoid without exoskeleton. (**b**) is the results of sEMG signals of biceps and deltoid with exoskeleton.

**Figure 11:**
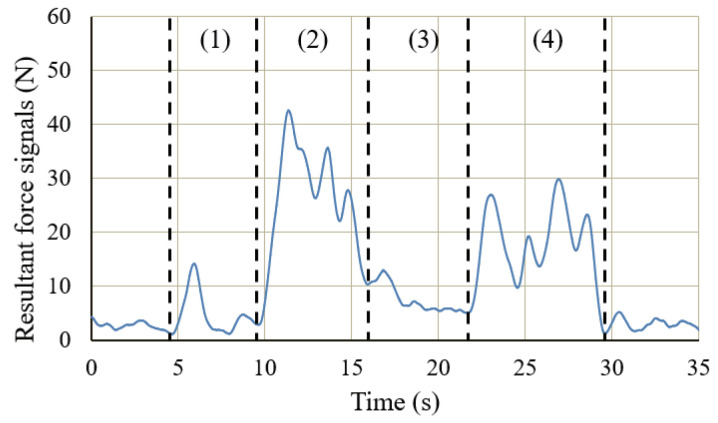
Resultant force signals for carrying a load to the 1.2 m high for subject A.

**Figure 12:**
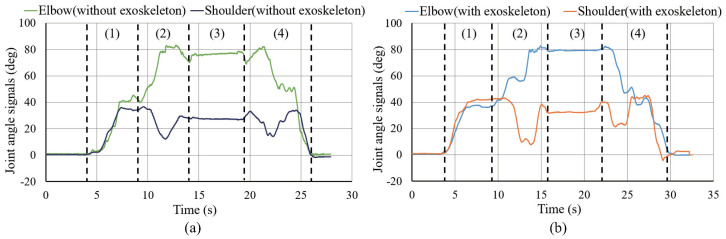
Joint angle signals for carrying a load to the 1.2 m high for subject A. (**a**) is the results of joint angle signals of elbow and shoulder without exoskeleton. (**b**) is the results of joint angle signals of elbow and shoulder with exoskeleton.

Figure 10 shows the changes in the sEMG signals during the carrying process. Figure 11 shows the resultant force signals of the force components ($f_{z}$ and $f_{y}$ in Figure 7) that were recorded by the force sensor on the exoskeleton. As the carrying work was performed on the plane of the flexion/extension, the force $f_{x}$ in Figure 7 was very small. Figure 12 shows the angle signals of the shoulder and elbow joints in the flexion/extension direction that were recorded by the motion capture system.

We have compared the measurement results of subject A. By comparting the results between the sEMG signals (Figure 10) and the joint angle signals (Figure 12) for subject A, which results in a weak change of the sEMG signals during the first stage. However, no noticeable changes were observed when the exoskeleton was not worn. During the third stage, although the heavy object was placed on the shelf, the arms had not been lowered yet, such that the sEMG signals represented the state of supporting the weight of the arms. On the other hand, the results of resultant force signals (Figure 11) and joint angle signals (Figure 12b) during the exoskeletal control show that the resultant force signals changed when the subject controls the exoskeleton to move together. The resultant force signals are changeless in the first phase because of absent load, but become noticeable in the second and fourth phases because it is the force required by the wearer to lift and lower the load, i.e., the force of the wearer on the exoskeleton. Finally, by comparing the results of sEMG signals with exoskeleton and without exoskeleton (Figure 10), the latter is significantly diminished. This indicates that the exoskeleton provides a real-time power assistance based on the force of the wearer. Additionally, weakening of the sEMG signals reflects the effectiveness of power assistance of the exoskeleton.

The second and fourth stages corresponded to the actions of lifting and lowering the heavy object, which bore the greatest load during the carrying process. Therefore, Equations (6) and (7) were used to calculate the results of the average sEMG signal and the average resultant force signal in Table 4 at these two stages. $t_{s}$ and $t_{e}$ are the start time and end time of these two stages, respectively:

(6) $$sEMG_{mean}=\frac{1}{t_{e}-t_{s}}\int_{t_{s}}^{t_{e}}\left|sEMG\right|dt$$

(7) $$Force_{mean}=\frac{1}{t_{e}-t_{s}}\int_{t_{s}}^{t_{e}}\sqrt{f_{z}^{2}+f_{y}^{2}}dt$$

According to the experimental results of subject A shown in Table 4, the average resultant force measured by the force sensor was 24.2 N when a heavy object was lifted, and it was 17.4 N when the object was put down. In comparison to the signals obtained in the case when the subject was not wearing the exoskeleton, the average sEMG signals acquired from the subject’s biceps and deltoid muscles during the lifting action when he or she was wearing the exoskeleton had dropped by 36.7% and 41.6%, respectively. Meanwhile, the signals acquired during the lowering action had dropped by 43.9% and 40.0%, respectively.

Table 4 also shows the experimental results for the other two subjects (B and C). For subject B, the average resultant forces of lifting and lowering actions were 24.7 N and 13.6 N, respectively. Their corresponding average sEMG signals were dropped by 40.7% and 47.2% for the biceps, and by 50.0% and 33.8% for the deltoid muscles. For subject C, the average resultant forces of lifting and lowering actions was 23.6 N and 15.8 N, respectively. The corresponding average sEMG signals from the biceps and deltoid muscles were dropped by 33.3% and 43.4% during the lifting action, and by 40.8% and 36.1% during the lowering action.

The average resultant force of the three subjects wearing the exoskeleton was less than 24.7 N during carrying an object of 10 kg. For the 10 kg object (each hand was considered to bear 5 kg (=49.0 N)), the exoskeleton had borne more than 49.6% of the weight. In other words, it provided power assistance to the wearer and reduced the burden on the wearer’s arms, which is consistent with the 50% target value set in the control. In addition, the sEMG signals of the three subjects also decrease significantly. Since the sEMG signals directly reflects the load on subject’s joint, our results suggest that the power assistance of the exoskeleton is effective to reduce the burden on the wearer’s muscles.

### 4.3. Experiment of Carrying a Heavy Object to the 1.8 m High

To carry a heavy object to a height of 1.8 m, a greater range of motion is required. Furthermore, lifting an object over the head requires highly coordinated movement of the elbow and shoulder joints—that is, elbow joint extension (angle decrease) and shoulder joint flexion (angle increase). An experimental comparison was performed for the cases with and without the exoskeleton being worn.

**Figure 13:**
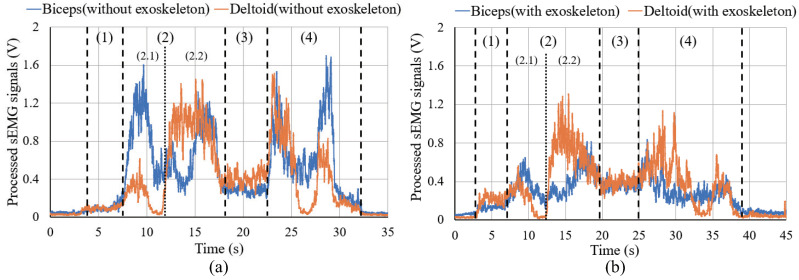
sEMG signals for carrying a load to the 1.8 m high for subject A. (**a**) is the results of sEMG signals of biceps and deltoid without exoskeleton. (**b**) is the results of sEMG signals of biceps and deltoid with exoskeleton.

**Figure 14:**
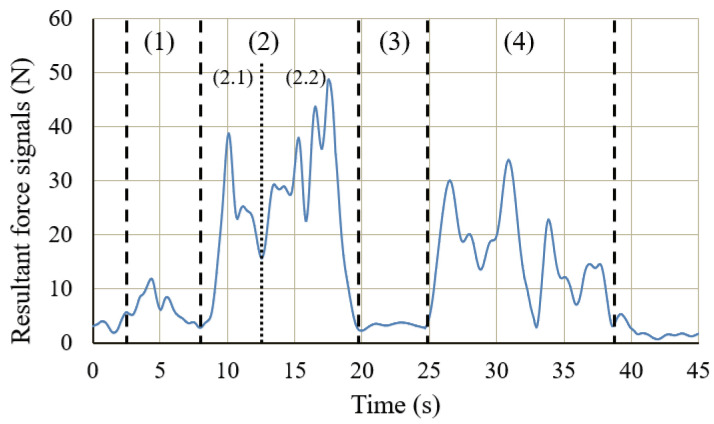
Resultant force signals for carrying a load to the 1.8 m high for subject A.

**Figure 15:**
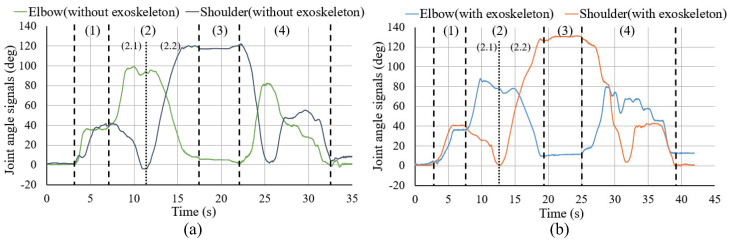
Joint angle signals for carrying a load to the 1.8 m high for subject A. (**a**) is the results of joint angle signals of elbow and shoulder without exoskeleton. (**b**) is the results of joint angle signals of elbow and shoulder with exoskeleton.

Figure 13 illustrates the changes in the sEMG signals during the carrying process. Figure 14 and Figure 15 display the resultant force signals recorded by the force sensor on the exoskeleton and the angle changes, respectively.

Unlike carrying an object to the 1.2 m high, carrying the object to the 1.8 m high requires a larger movement range, and the wearer naturally divides the movement of the second stage into two steps during the carrying process. First, (stage (2.1)), the wearer picks up the heavy object and approaches the shelf; subsequently (stage (2.2)), the wearer lifts the heavy object over his or her head in front of the shelf and proceeds to place it on top of the shelf.

As this movement requires a greater range of motion, the wearer naturally seeks a more effort-saving posture during the object-carrying process. For example, as shown in Figure 15 (stage (2.1)), the angle of the shoulder joint drops to $0^{{}^{\circ}}$ or even to a negative angle, where the burden on the shoulder joint is the smallest. Therefore, the sEMG signal of the deltoid muscle corresponding to the load on the shoulder joint was weakened significantly. According to the subjects’ narration, the target height in the 1.2 m experiment was relatively low, and they would want to lift the heavy object to this height and put it on the shelf faster. Therefore, the subjects did not deliberately seek a more effort-saving posture when approaching the shelf. Consequently, their shoulder joint angles were not reduced to $0^{{}^{\circ}}$, and the sEMG signals were not weakened significantly.

It can also be observed that the control of the exoskeleton as per our control law as shown in Equation (5) did not need to follow a specific trajectory. Instead, the exoskeleton provided power assistance on the basis of the action desired by the wearer.

The action of lifting the heavy object over the wearer’s head (stage (2.2)) is represented by the simultaneous increase in the shoulder joint angle and a decrease (extension) in the elbow joint angle, as shown in Figure 15. It can be observed from Figure 14 that, as the action progresses, the exoskeleton needs more output to support the heavy object; therefore, the resultant force increases. Similarly, the corresponding sEMG signals of the deltoid muscle in Figure 13 showed a significant increase in the muscle load during this action. In Figure 15, the joint angle changes observed in the cases with or without the exoskeleton were consistent, which also indicates that the use of the exoskeleton did not affect the wearer’s motion.

Although the action bacame more complicated, the results in Table 5 show that the average resultant force signals of subject A are 25.0 N and 16.5 N for the lifting and lowering actions, respectively. The average resultant force signals of lifting and lowering were 24.9 N and 17.5 N for subject B and 23.7 N and 16.0 N for subject C. For an object in weight of 10 kg (5 kg (=49.0 N) for each hand), the exoskeleton had borne more than 49.0% of the weight.

On the other hand, the average sEMG signals of biceps and deltoid muscles of subject A that had the power assistance from the exoskeleton were weakened by 37.3% and 36.1% during the lifting action and by 31.1% and 34.4% during the lowering action. For subject B, the average sEMG signals were weakened by 23.3% and 49.2% during the lifting and by 31.4% and 51.9% during the lowering. For subject C, the average sEMG signals were weakened by 22.8% and 55.8% during the lifting and 27.1% and 51.4% during the lowering. Above results also confirm that the power assistance of the exoskeleton works effectively.

Finally, in Figure 12 and Figure 15, the joint angle changes observed in the cases with or without the exoskeleton were consistent, which also indicates that the use of the exoskeleton did not affect the wearer’s motion.

## 5. Discussion

We have evaluated the exoskeleton with respect to backdrivability, workspace, and power-assist capability. Since the active joints of the exoskeleton are designed with a newly developed gear reducer and two-stage reduction, the backdrive torque is only 1.85 N·m, which is outstanding as compared with these backdrivable exoskeletons [7,32,33,34]. By comparing with the backdrivable exoskeleton with cable drive systems [7,32], our exoskeleton which uses the active joint is more compact and easier to wear. In addition, by comparing with the backdrivable exoskeleton with gear reducer (43.71:1) of [33] and (24:1) of [34], our exoskeleton achieves backdrive with the higher reduction ratio (300:1), which makes the exoskeleton more easier achieve greater output torque that is more suitable for power assistance. For the evaluation of the workspace of the exoskeleton, we have proposed a method of quantifying the exoskeleton’s workspace by calculating the reachable area of the wrist joint that presents more intuitively compared with the exoskeleton evaluated by the movable angle [7,8,9]. The measurement experiments have verified that the subjects with exoskeleton have achieved not less than 80% of the workspace of without exoskeleton, which indicates that the workspace of our upper-limb exoskeleton can meet the demands of daily use. For the power assistance function, the exoskeleton has been verified to be able to carry an object of 10 kg to a shelf at height of 1.2 m or 1.8 m depending on the wearer’s intention. The experimental results show that our exoskeleton can achieve more than a 49.0% power-assist ratio. These results are consistent with the 50% theoretical value as set in the control. Two power assistance experiments show that the developed exoskeleton can meet different scenarios of power assistance, including 1.2 m that is commonly used in daily life and 1.8 m that the exoskeletons applicable to 1.8 m are rarely mentioned in the current research. The above results show that the developed exoskeleton has excellent power assistance functions.

However, the comparison between the two power assistance experiments showed that the carrying process with the exoskeleton required a longer time than that without the exoskeleton (Figure 12 and Figure 15). This is because the high reduction ratio of the exoskeleton (300:1) had limited the maximum speed of the active joint. This problem will be addressed in our future research through an increase in the driving power and adjustment of the reduction ratio.

## 6. Conclusions

This study introduced the design of an upper-limb power-assisted exoskeleton. The active joint of the exoskeleton was provided with excellent backdrivability, which improves the comfort and safety of the exoskeleton. The DOF design and workspace of the exoskeleton was confirmed by motion capture system, which shows that the exoskeleton can fully meet the needs of daily life for upper limb mobility. Finally, we have verified the power-assist capability of the exoskeleton in actual carrying work.

## Figures and Tables

**Table 1:** Design parameters of each link length and joint angle of the exoskeleton.

Link	Length (m)	Joint	Rotation Range
$l_{1}$	0.05	$\theta_{1}$	$15^{{}^{\circ}}$ to $0^{{}^{\circ}}$
$l_{2}$	0.40	$\theta_{2}$	$\;0^{{}^{\circ}}$ to $120^{{}^{\circ}}$
$l_{3}$	0.09	$\theta_{3}$	$-20^{{}^{\circ}}$ to $100^{{}^{\circ}}$
$l_{4}$	0.09	$\theta_{4}$	$-30^{{}^{\circ}}$ to $170^{{}^{\circ}}$
$l_{5}$	0.15	$\theta_{5}$	$-90^{{}^{\circ}}$ to $90^{{}^{\circ}}$
$l_{6}$	0.10 to 0.25	$\theta_{6}$	$\;0^{{}^{\circ}}$ to $130^{{}^{\circ}}$
$l_{7}$	0.25		

**Table 2:** Comparison of the impact of wearing exoskeleton on the wearer’s shoulder workspace via motion capture.

Subject	Arm Length (m)	Workspace without Exoskeleton (m${}^{2}$)	Workspace with Exoskeleton (m${}^{2}$)	Coverage (%)
A	0.56	1.33	1.14	85.7
B	0.54	1.30	1.09	83.8
C	0.60	1.69	1.57	92.9
D	0.61	1.91	1.59	83.2

**Table 3:** Comparison of the impact of wearing exoskeleton on the wearer’s elbow workspace via motion capture.

Subject	Forearm Length (m)	Workspace without Exoskeleton (m${}^{2}$)	Workspace with Exoskeleton (m${}^{2}$)	Coverage (%)
A	0.25	0.24	0.22	91.7
B	0.23	0.21	0.18	85.7
C	0.27	0.25	0.23	92.0
D	0.28	0.27	0.24	88.9

**Table 4:** Summary of experiments involving carrying to the 1.2 m high.

Subject	Action	Status	${sEMG}_{mean}$ (V)		${Force}_{mean}$ (N)
			Biceps	Deltoid	Right-Hand
A	Lifting	without exoskeleton	0.60	1.01	-
		with exoskeleton	0.38	0.59	24.2
	Lowering	without exoskeleton	0.57	1.00	-
		with exoskeleton	0.32	0.60	17.4
B	Lifting	without exoskeleton	0.59	0.78	-
		with exoskeleton	0.35	0.39	24.7
	Lowering	without exoskeleton	0.53	0.68	-
		with exoskeleton	0.28	0.45	13.6
C	Lifting	without exoskeleton	0.63	0.71	-
		with exoskeleton	0.42	0.42	23.6
	Lowering	without exoskeleton	0.53	0.72	-
		with exoskeleton	0.30	0.46	15.8

**Table 5:** Summary of experiments involving carrying to the 1.8 m high.

Subject	Action	Status	${sEMG}_{mean}$ (V)		${Force}_{mean}$ (N)
			Biceps	Deltoid	Right-Hand
A	Lifting	without exoskeleton	0.59	0.72	-
		with exoskeleton	0.37	0.46	25.0
	Lowering	without exoskeleton	0.45	0.61	-
		with exoskeleton	0.31	0.40	16.5
B	Lifting	without exoskeleton	0.43	0.59	-
		with exoskeleton	0.33	0.30	24.9
	Lowering	without exoskeleton	0.35	0.54	-
		with exoskeleton	0.24	0.26	17.5
C	Lifting	without exoskeleton	0.57	0.95	-
		with exoskeleton	0.44	0.42	23.7
	Lowering	without exoskeleton	0.48	0.74	-
		with exoskeleton	0.35	0.36	16.0

## Abbreviations

The following abbreviations are used in this manuscript:

DOF	Degrees of freedom
sEMG	Surface electromyography
FSRs	Force-sensitive resistors
BLDC	Brushless direct current
3D	Three-dimensional
